# Cross-reactive broadly neutralizing antibodies: timing is everything

**DOI:** 10.3389/fimmu.2012.00215

**Published:** 2012-07-20

**Authors:** Zelda Euler, Hanneke Schuitemaker

**Affiliations:** Landsteiner Laboratory, Sanquin Research,Amsterdam, Netherlands; Department of Experimental Immunology, Center for Infection and Immunity Amsterdam, Academic Medical Center, University of Amsterdam,Amsterdam, Netherlands

**Keywords:** broadly neutralizing antibodies, cross-reactive broadly neutralizing antibodies, HIV-1, HIV-1 disease progression, humoral immunity

## Abstract

The recent surge of research into new broadly neutralizing antibodies in HIV-1 infection has recharged the field of HIV-1 vaccinology. In this review we discuss the currently known broadly neutralizing antibodies and focus on factors that may shape these antibodies in natural infection. We further discuss the role of these antibodies in the clinical course of the infection and consider immunological obstacles in inducing broadly neutralizing antibodies with a vaccine.

## INTRODUCTION

Thirty years after the discovery of HIV, UNAIDS estimates 34 million people are living with HIV and nearly 30 million people have died of AIDS-related causes since the first case of AIDS was reported on 5 June 1981 (www.unaids.org; Centers for Disease Control, 1981). While the number of new infections is declining globally and access to anti-retroviral (ARV) therapy for low- and middle-income countries has expanded to 6 million currently on treatment, the number of new HIV infections is still high at 7000 each day. However, recent advances with varying efficacy have been made in the prevention of HIV infection, which include adult male circumcision (Auvert et al., 2005; Bailey et al., 2007), ARV-based vaginal microbicides (Abdool et al., 2010), and pre-exposure prophylaxis (Grant et al., 2010). Moreover, the recent HPTN 052 trial has shown that ARV treated HIV-infected individuals are 96% less likely to transmit HIV to their uninfected partner (Cohen et al., 2011). However, ARV treatment would be a costly and complicated solution to halt the pandemic as the number of people infected still far surpasses the number of people treated and adherence to therapy is suboptimal. New preventative strategies are absolutely necessary, as several of currently known strategies pose challenges in implementation, not only in resource poor countries. Vaccines are by far the best strategy to prevent infection by all kinds of pathogens. For most vaccines against viruses, antibodies are a correlate of protection (Amanna et al., 2008; Plotkin, 2008).

Despite years of research, there is no vaccine that can establish protective immunity against HIV. The RV144 HIV vaccine trial was, however, the first to show some modest efficacy against acquisition of infection (Rerks-Ngarm et al., 2009). In this trial protection against infection was associated with antibodies directed against the second variable (V2) domain in the envelope of HIV. For a higher level or even complete protection against HIV infection, the upfront presence of neutralizing antibodies is considered to be most efficacious. To cover the huge variability of HIV-1, vaccine elicited neutralizing antibodies would need to be broadly reactive. However, broadly neutralizing antibodies were considered rare and moreover, attempts to design an immunogen capable of eliciting broadly neutralizing antibodies have failed so far. However, recent studies in the field have shown that broadly neutralizing antibodies may be more prevalent in HIV-1 infection than previously considered and these antibodies seem to have varying epitope specificities that could allow for multidirectional vaccine design. Here we review the role of broadly neutralizing antibodies in protection from HIV infection and HIV disease progression. In addition, we provide an overview of the specificities of the broadly neutralizing antibodies that are known to date and how this could translate in the optimal immunogen. Finally we discuss the conditions that may need to be fulfilled to elicit a protective humoral immune response.

## NEUTRALIZING ANTIBODIES TO HIV-1

**FIGURE 1:**
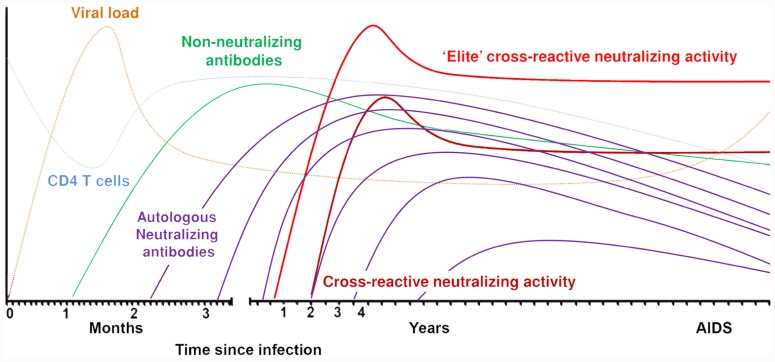
**Sequentially elicited antibodies to HIV-1 envelope over the course of the infection**.

The first detectable antibodies directed against the HIV-1 envelope appear around 12 days after infection. These antibodies are non-neutralizing, directed against the gp41 region, and mainly forming immune complexes (Tomaras and Haynes, 2009; **Figure 1**). Approximately 2 weeks later, antibodies are formed against the immunodominant gp120 region and these are also non-neutralizing. Neutralizing antibodies appear for the first time around 3 months post-seroconversion. These antibodies are mostly strain-specific and cannot neutralize heterologous viruses. Autologous neutralizing antibodies can rapidly select for escape variants of HIV-1 and do not seem to have a protective effect against progression to AIDS (Richman et al., 2003; Wei et al., 2003; Bunnik et al., 2008; Mahalanabis et al., 2009; van Gils et al., 2010). HIV-1 envelope is one of the most extensively glycosylated proteins found in nature (Myers and Lenroot, 1992). The glycans on the protein shield the conserved envelope domains from recognition by neutralizing antibodies. Also, as the glycans originate from the host cell machinery, they are usually not recognized by the immune system as foreign. Removal of glycosylation sites within or around the variable domains made the virus more susceptible to antibody neutralization (Reitter et al., 1998; Binley et al., 2010). The virus can escape neutralizing antibodies through additional potential N-linked glycosylation sites (PNGS) in the viral envelope or through changed positions of PNGS. The envelope also contains hypervariable regions that can change in length or amino acid sequence. HIV-1 variants with these changes can be rapidly selected under neutralizing antibody pressure if these changes coincide with an increased resistance to circulating neutralizing antibodies. Broadly neutralizing antibodies are directed against conserved regions of the virus and therefore capable of neutralizing a large variety of viruses from different subtypes. As these broadly neutralizing antibodies generally neutralize the majority of recently transmitted HIV-1 variants (Binley et al., 2004; Bunnik et al., 2010; Euler et al., 2011), irrespective of number of PNGS and variable loop length, a vaccine should be able to elicit this type of antibodies.

## THE FIRST BROADLY NEUTRALIZING ANTIBODIES

**FIGURE 2:**
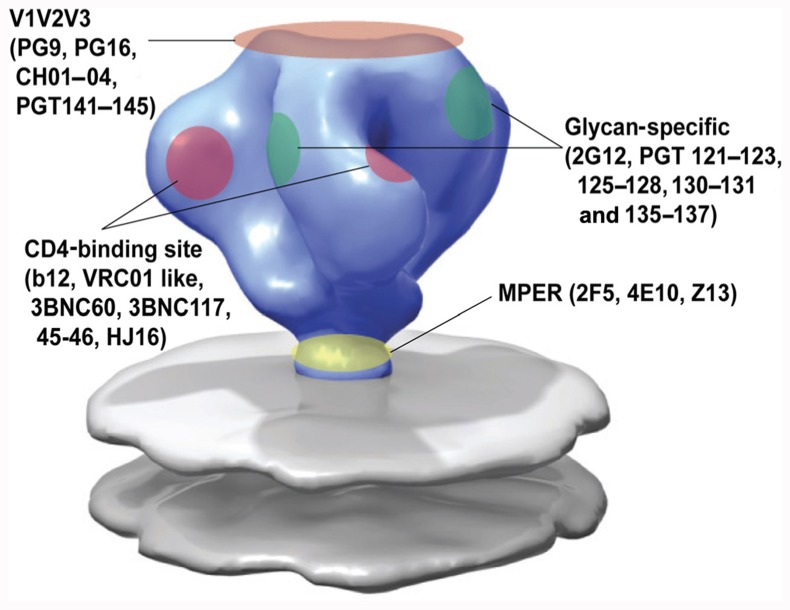
**Epitope specificity of known broadly neutralizing antibodies to HIV-1 envelope**. Adapted from White et al. (2010).

Prior to 2009, the presence of broadly neutralizing antibodies in HIV-1-infected individuals was considered rare. At that time, only four monoclonal antibodies (MAbs) had been isolated that could potently neutralize a majority of primary HIV-1 strains (**Figure 2**). MAb b12 was the first broadly neutralizing antibody and obtained from the bone marrow of an asymptomatic HIV-infected man (Burton et al., 1994; Kessler et al., 1997; Saphire et al., 2001). More specifically, it was isolated from a phage display library in which heavy and light chains are recombined *in vitro* to Fab fragments, and subsequently modified into complete IgG molecules. This may imply that the b12 antibody had not existed *in vivo* in the patient but only emerged due to *in vitro* recombination of heavy and light chains. The epitope on HIV-1 that is recognized by b12 lies in the CD4-binding site of the envelope. Crystal structures have revealed that the contact surfaces of b12 and CD4 on the viral envelope are considerably overlapping (Zhou et al., 2007), which could explain the neutralizing activity. However, overlap with the CD4-binding site alone is not sufficient for neutralization as the footprint for b12 on the CD4-binding site in Env overlaps with the footprints of antibodies b3 and b6 which are highly related to b12 but non-neutralizing. Antibodies b3 and b6 bind beyond the neutralizing face, into the non-neutralizing face and are unlike b12, not able to bind to trimeric Env (Pantophlet et al., 2003).

At the time, the only other broadly neutralizing antibody that was directed against gp120 was 2G12. This antibody has a very unusual dimer conformation and is directed against mannose structures on gp120, which results from post-transcriptional modifications by the host cell (Trkola et al., 1996; Sanders et al., 2002; Scanlan et al., 2002; Doores et al., 2010). Two of the other first identified broadly neutralizing MAbs, 4E10 and 2F5, were not directed against gp120 but instead against the membrane proximal region (MPER) in gp41 on the viral spike (Ofek et al., 2004; Cardoso et al., 2005; Zwick et al., 2005; Pejchal et al., 2009). These antibodies can only bind to their epitope in the context of the lipid bilayer of the membrane (Alam et al., 2007, 2009). Overall mAbs b12, 2G12, 2F5, and 4E10 neutralize approximately 35, 21, 43, and 56% of viral variants, respectively (Binley et al., 2004). Interestingly, there was an inverse correlation between breadth and potency of these antibodies, with b12 being potent, but not as broad, while 2F5 and 4E10 neutralized the majority of strains but with limited potency. Even among HIV-1 variants that were isolated from recently infected individuals, a relatively large number of neutralization resistant viruses were found (Quakkelaar et al., 2007; Euler et al., 2011) already suggesting that vaccine elicited antibodies would need to be better than these four antibodies to be efficacious. This and the unusual characteristics and rarity of b12, 2G12, 4E10, and 2F5 antibodies raised the quest to screen large cohorts for cross-reactive neutralizing activity (CrNA), in order to find broadly neutralizing antibodies with new epitope specificities that could be used for the design of immunogens capable of eliciting more potent CrNA in a vaccine setting.

## THE SEARCH FOR HIV-1-SPECIFIC CrNA

Large scale screening for CrNA in patient sera has intensified over the past few years in several cohorts across the world, that harbor individuals infected with different HIV-1 subtypes. Screening methods vary between different cohorts, especially with regards to the *in vitro* neutralization assays and the virus panels used, which could accurately define CrNA. Most widely used are a multiple-round peripheral blood mononuclear cell (PMBC)-based assay that requires primary virus isolates and a single-round infection assay using a reporter cell line, such as TZM-bl, that requires pseudoviruses produced by 293T cells. The neutralization sensitivity of viruses does vary between these two assays (Polonis et al., 2008, 2009; Fenyo et al., 2009; Rusert et al., 2009). However, there is a positive correlation between the PBMC- and TZM-bl-based assay in the identification of individuals with CrNA (van Gils et al., 2009). CrNA is defined as the ability of patient sera to neutralize the majority of viruses from different subtypes, although the exact cut off for CrNA varies per study. This may also explain, as least in part, the variation in prevalence of CrNA between cohorts, which varies from 10 to 33% (Beirnaert et al., 2000; Binley et al., 2008; Sather et al., 2009; Simek et al., 2009; van Gils et al., 2009; Doria-Rose et al., 2010; Euler et al., 2010; Medina-Ramirez et al., 2011; Mikell et al., 2011). In one of the largest studies, in which ~1800 HIV-1-infected people from Australia, Rwanda, Uganda, the United Kingdom, and Zambia were screened for CrNA on various virus panels, a mathematical algorithm was devised to reduce to amount of viruses that could predict the presence of CrNA (Simek et al., 2009). This large study also for the first time described the phenomenon of “elite neutralizers” (Simek et al., 2009) who in their serum have an average geometric mean IC_50_ titer of over 500, generally representing the top 1% of individuals with CrNA in serum within a cohort. This phenotype is independent of the clinical status of a person. CrNA in serum is not particular to individuals with an HIV-1 infection, as three recent studies showed that individuals with an HIV-2 infection have even more potent CrNA than individuals infected with HIV-1 (de Silva et al., 2012; Kong et al., 2012; Ozkaya et al., 2012). Identification of individuals with potent CrNA allows for the discovery of more broadly neutralizing antibodies with new epitope specificities based on which vaccine immunogens can be designed (Stamatatos et al., 2009).

## CrNA IN THE CLINICAL COURSE OF HIV-1 INFECTION

Broadly neutralizing antibodies are directed against conserved regions of the virus which may explain why these antibodies can neutralize a vast array of different HIV-1 strains from different subtypes. Based on this, it was plausible to think that broadly neutralizing antibodies would be capable of neutralizing the autologous virus strain *in vivo*, thereby reducing viral load and halting disease progression. As the first broadly neutralizing monoclonal antibody, b12, was isolated from an asymptomatic seropositive individual (Burton et al., 1991), it was thought that these antibodies were indeed able to protect from disease progression *in vivo*. However, after several screenings on long-term non-progressors (van Gils et al., 2009; Doria-Rose et al., 2010), elite controllers (Pereyra et al., 2008) and progressors (van Gils et al., 2009; Doria-Rose et al., 2010), there was no evidence that long-term non-progressors had better CrNA as compared to progressors. Indeed, recent screenings of entire cohorts of HIV-1-infected individuals, both men who have sex with men (MSM) with subtype B infections as well as heterosexually infected women with HIV-1 subtype C, did not show an association between the presence of CrNA and a prolonged asymptomatic phase in the course of HIV-1 infection (Piantadosi et al., 2009; van Gils et al., 2009; Euler et al., 2010; Gray et al., 2012). In a study with both long-term non-progressors and progressors, in which the prevalence of CrNA in serum was similar in both groups, neutralizing activity against autologous HIV-1 variants faded over time due to viral escape (**Figure 1**), explaining at least in part the absent effect of potent CrNA on the clinical course of infection (van Gils et al., 2010). Interestingly, CrNA against heterologous virus variants was preserved over the course of infection, despite escape of autologous virus. With the knowledge that CrNA generally takes 2–4 years to develop (**Figure 1**; Mikell et al., 2011; Euler et al., 2012; Gray et al., 2012) one could argue that the neutralizing humoral immune response simply comes too late to have an impact on disease course. Indeed, prior to the development of these antibodies the immune system may have been irreversibly damaged (Levesque et al., 2009), although obviously the ability of the patient to still develop CrNA argues against this. Even an elite neutralizer with potent CrNA from month 9 post-seroconversion onwards, that even further increased in breadth and potency until 3 years post-seroconversion, progressed to full blown AIDS within 7.5 years post-seroconversion (Euler et al., 2012). Interestingly, in this individual in particular, but also in other individuals with CrNA, an early diversification of the viral quasispecies was observed, which was not observed in individuals who did not develop CrNA. This may imply that CrNA provides extra immune pressure on the virus to escape and diversify as compared to autologous neutralizing activity that lacks cross-reactivity.

## PROTECTION IN MONKEYS BY BROADLY NEUTRALIZING ANTIBODIES

Despite the lack of protection against disease progression in individuals who naturally made broadly neutralizing antibodies *in vivo*, it cannot be excluded that these antibodies can protect against acquisition of infection when present at the time HIV-1 is encountered. Several studies in non-human primates have shown that passive immunization can confer protection against SIV or SIV/HIV hybrid virus (SHIV) infection in macaque models (Mascola et al., 1999, 2000; Parren et al., 2001; Veazey et al., 2003; Hessell et al., 2009a). Even at a relatively low antibody dose, b12 and 2G12 conferred protection in a repeat challenge model (Hessell et al., 2009a,b). Passive immunizations with antibodies b12, 2G12, and 2F5 also protected newborn macaques from oral SHIV challenge (Baba et al., 2000; Hofmann-Lehmann et al., 2001). Furthermore, vector mediated delivery of genes expressing broadly neutralizing antibodies in a humanized mouse model conferred protection against subsequent high dose viral challenges (Johnson et al., 2009; Balazs et al., 2012). This suggests that pre-existing humoral immunity may be able to prevent HIV-1 infection. Indeed, in a recent study, macaques that were vaccinated with MVA and/or adeno viral vectors expressing SIV Gag, Pol, and Env, partial protection against acquisition of the highly neutralization resistant SIV_MAC251_ challenge correlated with the presence of Env-specific binding and tier 1 neutralizing antibody titers prior to vaccination, specifically with an anti-V2 antibody response in the animals (Barouch et al., 2012).

## NEXT GENERATION BROADLY NEUTRALIZING ANTIBODIES

The screening for patients with CrNA in serum has intensified and so have the methods to isolate the broadly neutralizing antibodies from these patients. Several high-throughput techniques have been implemented (as reviewed by Moir et al., 2011) which has resulted in new antibodies that can neutralize the majority of HIV-1 strains tested, with a potency of up to four orders of magnitude to the previously identified broadly neutralizing antibodies (**Figure 2**).

### EPITOPE SPECIFICITIES OF BROADLY NEUTRALIZING ANTIBODIES

#### CD4-binding site

The host CD4 molecule is the major receptor for HIV-1. Attachment of Env gp120 to CD4 on the target cell initiates infection. Therefore, antibodies directed against the CD4-binding site on the gp120 envelope molecule are crucial to prevent binding of the virus to its host cell. In one approach, B cells that produced antibodies directed to the CD4-binding site of HIV-1 were isolated with a probe with a resurfaced stabilized core 3 (RSC3) in which all areas except the CD4-binding site of the virus were altered or masked, making those unfavorable for recognition (Zhou et al., 2010). With this method the MAbs, VRC01, VRC02, VRC03, and VRC-PG04, were cloned which bind specifically to the CD4-binding site, mimicking the binding between HIV-1 and the CD4 receptor (Wu et al., 2010, 2011; Zhou et al., 2010) with several overlapping binding sites. In a similar approach by Scheid et al. (2011), several highly active agonistic CD4-binding site directed antibodies (HAADs) were isolated from four unrelated elite neutralizers with a method that was described earlier (Scheid et al., 2009b). In this approach, single B cells were sorted with a probe that binds to a HIV gp120 core glycoprotein which was stabilized in the CD4-bound conformation and lacking the V1, V2, and V3 region (2CC core). Subsequently the corresponding heavy and light chains from these sorted B cells were cloned and expressed in cells that secreted the antibodies. Unlike the RSC3, the 2CC also binds to antibodies specific for the CD4-induced co-receptor binding site. To amplify the immunoglobulin gene, primers were selected further upstream from the highly variable complementary determining region 3 (CDR3) to circumvent the problem of the unusually high somatic mutations of the broadly neutralizing antibodies, as discussed below. With this method, 576 new antibodies were cloned, of which some were on par or even more potent than VRC01, including the antibodies 3BNC117, 3BNC60, and NIH45–46. These antibodies strongly resemble VRC01 both in crystal structure and in sequence convergence, including 10 of the contact residues between VRC01 and the HIV spike. In an attempt to increase the potency and breadth of these antibodies, based on structures-based design, Diskin et al. (2011) made a single substitution in the CDR2 of the heavy chain of NIH45–46 to create NIH45–46^G54W^, which increased the contact with the gp120 bridging sheet in turn improving the potency by an order of magnitude.

#### Quaternary antibodies against the variable loops in the viral envelope

In another large scale study, the approach of non-specific high-throughput memory B cell culture was taken (Walker et al., 2009, 2011). In this system activated B cells are cultured at near clonal density and the secreted antibodies are subsequently tested for neutralizing activity against a panel of HIV-1 variants. The corresponding heavy and light chain from B cells that secreted broadly neutralizing antibodies could then be reconstituted. With this technique, antibodies against novel epitopes of the virus were identified. Two such antibodies are PG9 and PG16, which are directed against an epitope that is preferentially expressed on the trimeric HIV Env protein across conserved regions of the variable loops of gp120 (Walker et al., 2009; Pancera et al., 2010). Since then, the antibodies CH01–04 (Bonsignori et al., 2011) and PGT141–145 (Walker et al., 2011) have been identified that target this same region. The recent crystal structure of gp120 V1V2 with PG9 revealed that this antibody as well as CH04 and PGT145 share a common mode of glycan penetration by extended anionic loops (McLellan et al., 2011). Although the epitopes of these antibodies are present in the monomeric structure, they are sensitive to the orientation of the viral spike and bind with a higher affinity to the trimeric form of gp120.

#### Glycan dependant antibodies

The non-specific high-throughput memory B cell culture system was performed with the B cells of four elite neutralizers, which resulted in the isolation of a new class of broadly neutralizing antibodies, termed the PGT series. These antibodies are directed against mannose structures and appear to be 10- to 100-fold more potent than other antibodies (Walker et al., 2011). Crystal structures of fab PGT128 together with a fully glycosylated gp120 outer domain showed that the antibody penetrates the glycan shield and recognizes two conserved glycans on positions N301 and N332, as well as a short β-strand segment of the V3 loop (Pejchal et al., 2011). The glycosylation sites which often shield the conserved sites of envelope against neutralizing antibodies, in this case render the virus more sensitive to neutralization.

### EPITOPE MAPPING

In order to find even more potentially conserved regions within the virus envelope, the undertaking of extensive epitope mapping of broadly neutralizing activity in sera of patients with CrNA has revealed various antibody specificities. There appears to be a difference in the number of antibody specificities between individuals with CrNA that is not so potent, and the so-called elite neutralizers (Simek et al., 2009). Previous studies on sera from individuals with CrNA with lower potency than observed in elite neutralizers showed that multiple epitope specificities exist within each infected individual (van Gils et al., in preparation; Dhillon et al., 2007; Li et al., 2007; Binley et al., 2008; Scheid et al., 2009a; Sather and Stamatatos, 2010; Tomaras et al., 2011). Conversely, CrNA in elite neutralizers was restricted to one or two epitope specificities per individual (Walker et al., 2010). Although the specificity was restricted within an individual, epitope specificity varied between individuals with elite neutralizing activity. Major antibody targets were associated with conserved regions of the V1, V2, and V3 loops as defined by the broadly neutralizing antibodies PG9 and PG16, as well as with an epitope overlapping the CD4-binding site. Identifying individuals with new epitope specificities could narrow the number of individuals from whom to isolate new broadly neutralizing antibodies.

## FACTORS INFLUENCING BROADLY NEUTRALIZING ANTIBODY FORMATION

A better understanding of how CrNA develops during the natural course of infection in some individuals will give valuable clues for vaccine design. CrNA is positively correlated with viral load, especially during the primary stage of infection (Piantadosi et al., 2009; Sather et al., 2009; van Gils et al., 2009; Doria-Rose et al., 2010; Mikell et al., 2011; Gray et al., 2012). This most likely implicates that sufficient antigen is required to stimulate the neutralizing humoral immune response. Surprisingly, CrNA is also associated with lower CD4 T cell counts early in infection (Euler et al., 2010; Gray et al., 2012). One could argue that these low CD4 T cell counts are probably just a consequence of the high viral load. However, the association was even observed for pre-seroconversion CD4 T cell counts (Euler et al., 2010). Furthermore, MSM showed more potent CrNA than injecting drug users (IDU; Euler et al., in preparation) which could be attributed to the presence of women in the cohort. Women had higher CD4 T cell counts at setpoint, which was the only independent predictor for the presence of CrNA in the Amsterdam Cohort studies.

As intact CD4 helper cells are necessary for class switching and multiple rounds of somatic hypermutation, this seems counterintuitive. Interestingly, in a lymphocytic choriomeningitis virus (LCMV) mouse model, lower CD4 T cell counts resulted in less HIV-1-induced polyclonal B cell activation, which in turn boosted virus-specific antibodies (Recher et al., 2004; Lang et al., 2007). Moreover, αβ T cell deficient mice still develop normal germinal centers and class-switched antibodies, in which case γδ T cells might be able to provide B cell “help” (Wen et al., 1996; Mbow et al., 2001; Brandes et al., 2003). It is tempting to speculate that only B cells with highest affinity will get sufficient help under conditions that CD4 cell counts are limited, thus contributing to the outgrowth of B cells with even higher affinity for their epitope.

Although CrNA was associated with overall lower CD4 T cell levels prior to infection and during acute infection, there was a positive association with the fraction of CD4 T cells expressing high levels of PD-1 (Mikell et al., 2011). PD-1 is expressed on follicular T helper cells and plays a crucial role in the formation of germinal centers and the proliferation and survival of circulating plasma cells (Good-Jacobson et al., 2010).

In a recent genome wide association study (GWAS) on the development of CrNA, we have found several single nucleotide polymorphisms (SNPs) in the gene region of MHC class I chain-related protein A (MICA) on chromosome 6 that were associated with the development of CrNA at 3 years post-serconversion (Euler et al., submitted for publication). The association of these SNPs with CrNA was strong but did not reach genome wide significance after correction for multiple testing and confirmation of this finding is required. Interestingly, previous studies have shown strong associations between SNPs on chromosome 6 and viral load set point (Fellay et al., 2007; Dalmasso et al., 2008; Pelak et al., 2010) which as mentioned above by itself has been associated with CrNA in several studies (Piantadosi et al., 2009; Sather et al., 2009; van Gils et al., 2009; Doria-Rose et al., 2010; Mikell et al., 2011; Gray et al., 2012). However, the association between MICA and CrNA was only partially decreased after correction for viral load as a covariate, indicating that other yet unknown mechanisms independent of viral load could play a role in the development of CrNA.

Other covariates such as HLA class II alleles, risk group, history of using ARVs, race, ethnicity, gender, and age were not associated with the development of CrNA (Doria-Rose et al., 2010; Euler et al., submitted for publication).

Obviously, characteristics of the HIV-1 variant that is transmitted can also have an impact on the induction of CrNA. In order to find viral genetic signatures associated with CrNA, Gnanakaran et al. (2010) screened sera from 69 individuals for CrNA and subsequently clustered them in a high and a low neutralizing potency group. Six signature positions in the Env sequences of their HIV-1 variants were associated with high or low neutralizing potency in serum. Five of these were in the CD4-induced co-receptor binding site of gp120. A similar approach in a subtype C-infected cohort showed that shorter V1–V4 regions from autologous viruses corresponded with higher neutralization against a heterologous viral panel (Rademeyer et al., 2007). In this study, Envs were isolated at the same time point at which CrNA was detected. At that point, HIV-1 may have already escaped from autologous neutralization (van Gils et al., 2010; Euler et al., 2012) and one could therefore argue that these Env sequences have signals for escape from CrNA, rather than signals that are specific for the induction of CrNA. HIV-1 variants isolated during the primary stage of infection with proven sensitivity to autologous serum with CrNA might be more relevant for the identification of signatures between individuals with and without CrNA. A more open viral envelope configuration might better expose certain conserved epitopes that can induce the formation of these antibodies. In a recent study we have revealed an association between CrNA in serum and the presence, in the first year of infection, of HIV-1 variants with envelopes that had a shorter V1 region and a lower NXS/NXT ratio, the latter indicating fewer PNGS (van den Kerkhof et al., in preparation). These viruses were still sensitive to autologous serum neutralization and may have been the drivers for eliciting CrNA in these patients.

## VACCINE ELICITED CrNA: IMMUNOLOGICAL OBSTACLES

The known broadly neutralizing antibodies have unique characteristics, which also complicates the induction of these antibodies. A majority of broadly neutralizing antibodies have a long CDR3 in their immunoglobulin heavy chains. Two such antibodies, PG9 and PG16, have an exceptionally long CDR3, which forms a type of “hammerhead” with which it interacts with HIV-1 Env. Interestingly, it appears that B cells with long CDR3 regions undergo negative selection at the naïve B cells stage (Shiokawa et al., 1999; Meffre et al., 2001). Polyreactive and self-reactive B cells are also negatively selected at the early B cell stages, yet in HIV-1 infection, several broadly neutralizing antibodies are polyreactive (Haynes et al., 2005), and up to 70% of memory B cells specific for HIV-1 Env from individuals with CrNA are thought to be polyreactive (Mouquet et al., 2010), defined by their ability to bind to multiple and distinct antigens on self-antigens and other non-viral antigens. It is thought that the low density of viral spikes on the membrane of HIV-1 causes antibodies to bind bivalently to both the gp160 trimer and non-HIV antigens resulting in higher affinity, but in turn also more polyreactivity (Klein and Bjorkman, 2010). In a study to test if affinity could enhance the neutralizing capacity of bivalent antibodies, bi-specific anti-HIV-1 antibodies were engineered that bind gp120 with one arm and gp41 with the other. These bi-specific antibodies were better neutralizers than their independent parental mono-specific antibodies (Mouquet et al., 2012). Mouquet et al. (2010) hypothesized that during the primary immune response to HIV-1, polyreactive B cells are positively selected due to their strong affinity, which is based on their bivalent binding, which is known to correlate with virus neutralization. It has been suggested that anti-gp41 antibodies formed during the acute infection are polyreactive due to gp41-specficic B cell stimulation of non-HIV-1 antigens prior to infection (Liao et al., 2011).

However, polyreactivity is no prerequisite for antibodies to neutralize HIV, as b12, 2G12, and VRC01 have very little polyreactivity (Haynes et al., 2005). Also the new broadly neutralizing PGT antibodies have very little polyreactivity, despite the fact that their epitope is from host origin (Walker et al., 2011).

Another unique characteristic of broadly neutralizing antibodies is the high degree of somatic mutations within the CDR3 region as compared to antibodies in other infections which typically accumulate only 5–15% changes in this region during affinity maturation (Wrammert et al., 2011). The degree of somatic mutations of the known broadly neutralizing antibodies ranges from 6% for 4E10 (McElrath and Haynes, 2010) to 19% for PG9 and PG16 (Walker et al., 2009) and to up to 32% for some of the CD4-binding site directed antibodies (Wu et al., 2010). Thus, the mutational pathways for creating such neutralizing antibodies could be more complex than those from other diseases (Dimitrov, 2010). *In vitro* reverted unmutated ancestor antibodies from HIV-1-specific broadly neutralizing antibodies have shown little or no reactivity with the epitopes of their progeny broadly neutralizing antibodies (Xiao et al., 2009; Mouquet et al., 2010; Pancera et al., 2010; Zhou et al., 2010; Bonsignori et al., 2011). This indicates that broadly neutralizing antibodies in general may need multiple rounds of somatic mutation and affinity maturation and that the currently known recombinant envelope glycoproteins are insufficient in promoting this process to achieve the desired mature antibodies from precursor naïve B cells. However, when Env was partially deglycosylated, the reverted unmutated ancestors of 2F5 and 4E10, could bind to these Envs, but not to the fully glycosylated Env (Ma et al., 2011). This suggests that germline B cell receptors have the capacity to bind to the relevant epitopes, but that this binding might be impeded or even prevented by glycan interference. Additionally, germline predecessors of broadly neutralizing antibodies have shown to be different between rhesus macaques and humans (Yuan et al., 2011) which might explain the limited success in the generation of these antibodies in animal models. Identifying antibodies that are intermediates in the pathways to maturation, but not yet broadly neutralizing, could help design conceptually novel vaccine immunogens (Dimitrov, 2010) as immunogens that can induce these intermediate antibodies could guide the immune system to mature in the direction of highly mutated broadly neutralizing antibodies.

Current strategies to unravel the pathway of affinity maturation that leads to the formation of broadly neutralizing antibodies includes 454 pyrosequencing of B cells of HIV-1 individuals to identify their antibody repertoire or antibodyome (Dimitrov, 2010; Bonsignori et al., 2011; Wu et al., 2011; Zhu et al., 2011). In the study by Wu et al. (2011) the authors identified new VRC01-like antibodies by means of sequence data obtained from bulk PMBC, with high divergence from the inferred germline genes and sequence similarity to VRC01. They observed high structural convergence to the epitope-binding region as well as functional complementation between the heavy and light chains of all these antibodies, suggesting that these antibodies could mature in the same way and therefore could predict the maturation of these antibodies. However, the question remains if such maturation pathways can be induced *in vivo*. In one recent study, in order to program the B cell response, the natural evolution within HIV-infected individuals was mimicked in an animal model by sequential immunization with different Env proteins. Compared to animals that received a cocktail of Envs and only one clonal Env, the sequential immunizations resulted in the highest heterologous neutralization titers (Malherbe et al., 2011), suggesting that the immune response can be focused towards a broader neutralizing response. These results can imply that vaccination against HIV-1 may need multiple long-term boosting with different antigens. Indeed, an extra late stage boost in the RV144 trial could have potentially improved the efficacy in that study.

## NEUTRALIZATION SENSITIVITY OF HIV-1 OVER THE COURSE OF THE EPIDEMIC: SETTING THE BAR FOR VACCINE DEVELOPMENT

Recently, we have shown that HIV-1 has become more resistant to antibody neutralization over the course of the epidemic (Bunnik et al., 2010). This effect was most notable to the CD4-binding site directed antibodies b12 and VRC01 (Bunnik et al., 2010; Euler et al., 2011). This increased resistance to humoral immunity was associated with an extended and more heavily potentially glycosylated V1 loop (Bunnik et al., 2010). Moreover, the exchange of the V1V2 region from viruses isolated from individuals who seroconverted in the beginning of the epidemic with the V1V2 regions from HIV-1 variants from contemporary seroconverters, rendered the viruses more sensitive to neutralization (van Gils et al., 2011). This implicates that the V1V2 loops shield vulnerable epitopes, especially epitopes in the CD4-binding site, from neutralizing antibodies.

Despite the increased neutralization resistance, currently circulating HIV-1 strains are sensitive to neutralization by multiple of the newly identified broadly neutralizing antibodies at low concentrations, in particular to Mabs PG9, PG16, VRC01 as well as PGT121 and the HAADS 3BNC117, 3BNC60, and NIH45–46 (Euler et al., 2011; Euler et al., unpublished data). In the face of a changing HIV-1 landscape over the course of 20 years, potent antibodies with exceeding neutralizing breadth are required to target multiple sites of the viral envelope in order to achieve sterilizing immunity. With the newly identified antibodies against mannose structures as well as new antibodies against the CD4-binding site with an up to 10-fold increase in neutralization potency compared to PG9, PG16, and VRC01 (Walker et al., 2011), contemporary circulating HIV-1 strains should be neutralized by at least one of the known broadly neutralizing antibodies. A vaccine that can elicit these specificities would therefore likely be efficacious even against currently circulating, more neutralization resistant HIV-1 variants.

## CONCLUSION

Many challenges remain in the development of an immunogen that is capable of eliciting broadly neutralizing antibodies. Besides the structural constraints on the immunogen, there are immunological barriers that need to be tackled, including the long affinity maturation that B cells seem to require before they can produce broadly neutralizing antibodies, and the risk of eliciting antibodies that have potential self-reactivity. If polyreactivity is a key feature of broadly neutralizing antibodies, then the chances of eliciting them with an envelope-based immunogen that is presented in the absence of a membrane context may be complicated or even impossible. The identification of even more broad and potently neutralizing antibodies against not previously identified epitopes will hopefully bring us ever more closer to the design of an immunogen that can overcome these challenges. Most circulating HIV strains are sensitive to the majority of broadly neutralizing antibodies known to date *in vitro*. However, *in vivo*, naturally developed broadly neutralizing antibodies have no effect on disease progression as the virus easily escapes. Therefore, an effectively neutralizing humoral immune response needs to be in place prior to encountering HIV in order to prevent infection. As soon as infection is established, the humoral immune response is not likely to play a role in controlling viral replication.

An HIV vaccine is not available but huge progress has been made in understanding what is required to neutralize HIV-1 and how this type of immunity may be elicited. Understanding the problem may pave the way to its solution.

## Conflict of Interest Statement

The authors declare that the research was conducted in the absence of any commercial or financial relationships that could be construed as a potential conflict of interest.
